# Crossmodal Links between Vision and Touch in Spatial Attention: A Computational Modelling Study

**DOI:** 10.1155/2010/304941

**Published:** 2009-10-22

**Authors:** Elisa Magosso, Andrea Serino, Giuseppe di Pellegrino, Mauro Ursino

**Affiliations:** ^1^Department of Electronics, Computer Science and Systems, University of Bologna, 40136 Bologna, 47023 Cesena, Italy; ^2^Department of Psychology, University of Bologna, 40127 Bologna, Italy; ^3^Centro Studi e Ricerche in Neuroscienze Cognitive, 47023 Cesena, Italy

## Abstract

Many studies have revealed that attention operates across different sensory modalities, to facilitate the selection of relevant information in the multimodal situations of every-day life. Cross-modal links have been observed either when attention is directed voluntarily (endogenous) or involuntarily (exogenous). The neural basis of cross-modal attention presents a significant challenge to cognitive neuroscience. Here, we used a neural network model to elucidate the neural correlates of visual-tactile interactions in exogenous and endogenous attention. The model includes two unimodal (visual and tactile) areas connected with a bimodal area in each hemisphere and a competition between the two hemispheres. The model is able to explain cross-modal facilitation both in exogenous and endogenous attention, ascribing it to an advantaged activation of the bimodal area on the attended side (via a top-down or bottom-up biasing), with concomitant inhibition towards the opposite side. The model suggests that a competitive/cooperative interaction with biased competition may mediate both forms of cross-modal attention.

## 1. Introduction

Our environment constantly provides us a large amount of information. An important goal for the brain is to filter out irrelevant information and to select only relevant events in order to guide behaviour. A basic mechanism for selecting information is to process stimuli from a limited portion of space; this function is mediated by *spatial attention*. Most research on spatial attention has been focused on purely unimodal situations [1–5]. Most studies show that responses to stimuli presented at the attended locations increased in comparison to those at the unattended locations, both at behavioural and electrophysiological level. Moreover, extensive theoretical and experimental work on the visual system [1, 3, 6–9] has suggested an influential hypothesis about the neural mechanisms underlying visuospatial attention, known as the *biased competition hypothesis*. The basic idea is that attention biases the competition between multiple stimuli in the visual field in favour of one stimulus, so that neurons encoding the attended stimulus win the competition and suppress the activity of the cells representing unattended stimuli. The competition among concurrent stimuli can be biased both voluntarily, when the subject dedicates more attentional resources to a given spatial position (endogenous or top-down spatial orienting), or reflexively, when an external stimulus cue suddenly appears at a given spatial location (exogenous or bottom-up spatial orienting) [10, 11].

Recently, the issue of spatial attention has been addressed from a cross-modal perspective. Indeed, an external event typically produces multimodal signals, stimulating different senses simultaneously (as when a visible object moves to touch the body, e.g.). Hence, attention needs to be coordinated across different sensory modalities, in order to select visual, auditory, and tactile information originating from the same object or event, meaning the same spatial location. Behavioural studies showed several cross-modal interactions in spatial attention, that involve different combinations of modalities (e.g., visual-acoustic, visual-tactile). Both *exogenous* and *endogenous* spatial orienting have been studied *crossmodally*[12–15]. In exogenous attention paradigms, the appearance of a single target is preceded by an abrupt “cue,” which is task-irrelevant and spatially nonpredictive (i.e., the cue location does not predict where the subsequent target stimulus will appear). Responses have been found to be faster and more accurate for targets that appear shortly afterwards on the same side as the cue than on the opposite side, even when the cue and target modalities are different; this suggests that attention is automatically captured towards the cue location not only within the cued modality but also in other modalities. Cross-modal links also exist in the more active, endogenous, form of attentional orienting. When subjective expectations about the appearance of a target in one sensory modality direct attention towards a given side of space, responses are facilitated on that side compared to the opposite side, not only for targets in the attended modality but also for targets in a secondary modality.

A number of studies have investigated the neural correlates of crossmodal attentional interactions using event related potentials (ERP) [13, 16, 17] and hemodynamic measures [18, 19]. Some results support the idea that cross-modal links in spatial attention may reflect the activity of a supramodal system that controls attentional orienting in different modalities. Such account accords with recent neurophysiological findings of multimodal neurons, especially in the parietal and premotor cortex, that respond to multiple modalities in approximate spatial register [20–22]. However, the neural circuits and mechanisms implicated in cross-modal attentional links are not clearly identified yet, and important issues are still open. Among the others, it is not yet clear whether the conceptual framework of the biased competition hypothesis, which successfully accounts for different aspects of visual attention, may apply also for cross-modal attentional effects, and whether the two forms of attention (endogenous and exogenous) are mediated by the same or different neural mechanisms.

Recently, we have developed a neural network model that simulates visual-tactile integration in parietal and frontal regions, involved in the perception of the space near the body (peripersonal space) [23]. In particular, the network mimics the visuotactile representation of the peripersonal space around both hands and is able to account for several results both in healthy subjects and in neuropsychological patients. Moreover, the model has been used to investigate the resizing of perihand space following tool use [24].

The aim of this study is to use an adapted version of our network to investigate the neural bases of visual-tactile interactions in endogenous and exogenous spatial attention.

## 2. Model Description

### 2.1. Qualitative Model Description

The present paper aspires to simulate and interpret—by using an appropriate neural network model—experimental results of visual-tactile links in attention. All experimental data, which the model aspires to reproduce and to which simulation results are compared, are taken from previous studies in literature performed by other researchers (in particular, Spence et al. [13, 15]); that is, the present study is entirely a theoretical study, not including any experimental design and participants. In particular, the proposed model aims at reproducing only results of *covert orienting *of exogenous [13] or endogenous attention [15]; covert orienting of attention involves purely internal attentional shifts, without any receptor shift (absence of eyes, head or hands movements). In the above quoted studies, participants (healthy subjects) adopted a fixed “default” posture, with eyes and head facing forward (participants were required to fixate a central point), and the hands resting symmetrically each in its own hemispace; tactile and visual stimuli were applied only on the hands. The tactile stimuli were delivered via tactile stimulators attached to or in contact with the hands; visual stimuli were delivered via led positioned in close proximity to the tactile stimulators; a central led served as fixation point [13, 15]. Tactile and visual targets had a duration of several tens of milliseconds (200 milliseconds); in exogenous attention paradigm [13], the cue preceding the target had a shorter duration (few tens of milliseconds).

**Figure 1:**
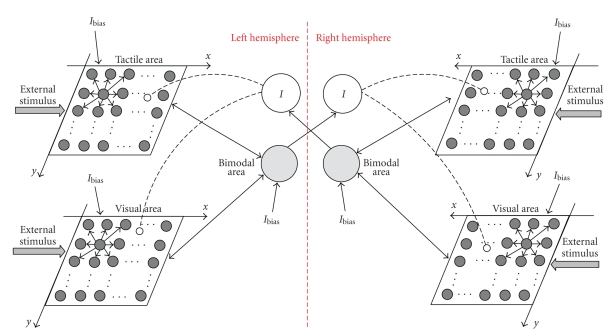
Schematic diagram describing the network. Continuous lines represent excitatory connections (lines with arrows in both direction denotes the presence of both feedback and feedforward connections); dashed lines represent inhibitory connections. *I* denotes inhibitory interneurons. *I*_bias_ is a top-down bias input mimicking endogenous attention conditions; its value may be selectively modified in each of the six areas.

The model has been conceived to reproduce and interpret the above described experimental studies of covert attention [13, 15]. Accordingly, the model includes two subnetworks, one per hemisphere, each referred to the contralateral hand; moreover, owing to the assumption of fixed postural conditions, we did not included any postural signal in the model and the only external inputs are tactile and visual stimuli (see Figure 1). We hypothesized—with reasonable approximation—that the hand of an adult subject is 10 cm wide and 20 cm length, thus roughly covering a surface of 10 cm × 20 cm.

The single subnetwork embodies three areas of neurons, which communicate via synaptic connections. The two upstream areas are bidimensional matrices of unimodal neurons: neurons in one area respond to tactile stimuli on the contralateral hand (*tactile area*); neurons in the other area respond to visual stimulation on the same hand *(visual area)*. Each unimodal neuron has its own *receptive field* (RF), reproduced by means of a Gaussian function, through which receives external stimulation. In both areas, the RFs are in hand-centered coordinates and are arranged according to a topological organization, so that each area maps the external (visual or tactile) space in an orderly manner (proximal neurons respond to stimuli coming from proximal positions in the space). In particular, we assumed that the RFs in both unimodal areas are arranged at a distance of 0.5 cm along both the *x* and *y* directions; this choice is arbitrary and has been made to represent the hand surface of 10 cm × 20 cm by using a limited number of neurons (20 × 40), in order to maintain model computational weight within acceptable limits. However, each neuron in the model should not be considered representative of a single cell, but rather as a pool of cells with the RF approximately located in the same position. It is worth noting that in the present model, the visual area embodies the same number of neurons as the tactile one (20 × 40 neurons), exactly coding the visual space *on* the hand (10 cm × 20 cm): indeed, in this study we consider only visual stimuli applied on the hand surface. Neurons within each unimodal area interact via *lateral synapses* with a Mexican hat arrangement (i.e., short range excitation and long range inhibition).

The unimodal tactile and visual neurons in each subnetwork send *feedforward synapses* to a third downstream multimodal area devoted to visual-tactile integration. For the sake of simplicity, we consider a single visual-tactile neuron, covering the entire hand. The feedforward synapses linking the unimodal (either visual or tactile) neurons to the downstream bimodal neuron have a uniform distribution; that is, their value is independent of the position of the unimodal neuron in the area.

The bimodal visual-tactile neuron within one hemisphere sends feedback excitatory synapses to the upstream unimodal areas within the same hemisphere. The feedback synapses have a uniform distribution, too.

The two subnetworks are reciprocally interconnected via inhibitory interneurons, realizing a competitive mechanism. The inhibitory interneuron in one hemisphere receives information from the bimodal neuron in the other hemisphere, through a synaptic connection characterized by a *pure delay*, to account for the interhemispheric transit time via the corpus callosum; then, the interneuron sends inhibition to the local unimodal areas. In the model, the inhibitory synapses from the interneuron to tactile and visual neurons have a uniform distribution.

Finally, unimodal and bimodal neurons within each hemisphere may receive a top-down bias input (*I*_bias_) mimicking attention conditions (see Section 2.3). We assumed *I*_bias_ = 0 in basal conditions.

The input-output relationship of each neuron (both unimodal, bimodal and inhibitory) includes a first-order dynamics and a static sigmoidal relationship with a lower threshold and an upper saturation. Each neuron in the network is normally in a silent state and can be activated if stimulated by a sufficiently high excitatory input.

### 2.2. Model Equations

In the following, the main equations of the model are presented. Since the overall network has a symmetrical structure, only equations for one hemisphere (the left one) will be reported. The superscripts *t*, *v*, and *m* will denote quantities referring to tactile, visual, and multimodal (i.e., bimodal) neurons, respectively; the superscript *g* will indicate quantities referring to the inhibitory interneuron; the superscripts *L* and *R* will distinguish the left and right hemisphere; the subscripts *ij* or *hk* will represent the spatial position of individual neurons.

#### 2.2.1. The Unimodal Neurons

The unimodal areas are composed of *N* ^*s*^ × *M* ^*s*^ neurons (*s* = *t*, *v*), with *N* ^*s*^ = 20, *M* ^*s*^ = 40. In both areas, the RFs of neurons are arranged at a distance of 0.5 cm along both the *x* and *y* directions.

By considering a reference frame rigidly connected with the hand (see Figure 1), the coordinates of the RF centre of a generic neuron *ij* are given by

(1) $$\begin{array}{l} x_{i}^{s}=i\cdot 0.5\;\text{cm}\;\left(i\;=\;1,2,\ldots ,N^{s}\right),\\ y_{j}^{s}=j\cdot 0.5\;\text{cm},\;\left(j=1,2,\ldots ,M^{s}\right), \end{array}\;s=t,v.$$

The receptive field of the unisensory neurons is described via a Gaussian function. For a generic neuron *ij*, the following equation holds:

(2) $$\Phi_{ij}^{s,L}\left(x,y\right)=\Phi_{0}^{s,L}\cdot \exp\;\left(-\;\;\frac{{\left(x_{i}^{s,L}-x\right)}^{2}+{\left(y_{j}^{s,L}-y\right)}^{2}}{2\cdot {\left(\sigma_{\Phi }^{s,L}\right)}^{2}}\right),\;\;\;\;\;\;s=t,v,$$

where *x*_*i*_ and *y*_*j*_ are the centre of the RF,* x* and *y* are the spatial coordinates, and Φ_0_^*s*, *L*^ and *σ*_Φ_^*s*, *L*^ represent the amplitude and standard deviation of the Gaussian function. According to this equation, a punctual external stimulus applied at the position *x*, *y* excites not only the neuron centred in that point but also the proximal neurons whose receptive fields cover that position.

The total input received by a generic neuron *ij* in the unimodal area *s* = *t*, *v* is given by five different contributions.

(a) The first contribution is due to the external stimulus, computed as the inner product of the stimulus and the receptive field:

(3) $$\begin{array}{ll} \varphi_{ij}^{s,L}\left(t\right) & =\int_{x}^{}\int_{y}^{}\Phi_{ij}^{s,L}\left(x,y\right)\cdot I^{s,L}\left(x,y,t\right)dx\;dy\\ & ≅\underset{x}{\sum }\;\sum_{y}\Phi_{ij}^{s,L}\left(x,y\right)\cdot I^{s,L}\left(x,y,t\right)\Delta x\Delta y,\;s=t,v, \end{array}\;$$

where *I* ^*s*,*L*^(*x*, *y*, *t*) is the external stimulus (tactile or visual) applied on the righthand (processed by the left hemisphere) at the coordinates *x*, *y* and at time *t*. The right-hand member of (3) means that the integral is computed with the histogram rule (with Δ*x* = Δ*y* = 0.2 cm).

(b) The second contribution is due to the lateral synapses linking the neuron with the other elements in the same area. This contribution is defined as

(4) $$\lambda_{ij}^{s,L}\left(t\right)=\overset{N^{s}}{\underset{h=1}{\sum }}\overset{M^{s}}{\underset{k=1}{\sum }}\Lambda_{ij,hk}^{s,L}\cdot z_{hk}^{s,L}\left(t\right),\;s=t,v.$$

*z*_*hk*_^*s*, *L*^(*t*) represents the activity of the *hk* neuron in the area *s *(*s* = *t*, *v*) of the left hemisphere. Λ_*ij*, *hk*_^*s*, *L*^ indicates the strength of the synaptic connection from the presynaptic neuron at the position *hk* to the postsynaptic neuron at the position *ij*. These synapses are symmetrical and are arranged according to a “Mexican hat” function.

(c) The third contribution is due to the feedback excitatory projections from the bimodal neuron, computed as

(5) $$\beta_{ij}^{s,L}\left(t\right)=B_{ij}^{s,L}\cdot z^{m,L}\left(t\right),\;s=t,v.$$

*z* ^*m*,*L*^(*t*) represents the activity of the bimodalneuron. *B*_*ij*_^*s*, *L*^ indicates the strength of the synaptic connection from the presynaptic bimodal neuron to the postsynaptic unimodal neuron *ij*; in the model the feedback synapses have a uniform distribution (i.e., *B*_*ij*_^*s*, *L*^ = *B*_0_^*s*, *L*^, for all *i*, *j*).

(d) The fourth contribution is due to the synapses from the inhibitory interneuron, given by

(6) $$\gamma_{ij}^{s,L}\left(t\right)=\Gamma_{ij}^{s,L}\cdot z^{g,L}\left(t\right),\;s=t,v.$$

*z* ^*g*,*L*^(*t*) represents the activity of the inhibitory interneuron, which depends on the visual-tactile information at the other hemisphere (see below). Γ_*ij*_^*s*, *L*^ is the strength of the synaptic connection from the interneuron to unimodal neuron *ij*; the inhibitory synapses have a uniform distribution, too (Γ_*ij*_^*s*, *L*^ = Γ_0_^*s*, *L*^, for all *i*, *j*).

(e) The fifth contribution is due to the attentional top-down bias input. We assumed that the attentional bias to one unimodal area acts as a constant input (*I*_bias_^*s*, *L*^) and affects all the neurons within the area.In basal conditions, the attentional bias input is set equal to zero in both unimodal areas of each hemisphere.

The total input is obtained by summing the four excitatory contributions (a), (b), (c), and (e) and subtracting the inhibitory contribution (d). Then, neuron activity (*z*_*ij*_^*s*, *L*^(*t*)) is computed from the total input through a first-order dynamics and a static sigmoidal relationship.

#### 2.2.2. The Bimodal Neuron

The overall input to a bimodal neuron in one hemisphere is the sum of two contributions.

(a) The first contribution is due to the inputs from neurons in the two unimodal areas via feedforward synapses, computed as

(7) $$\eta^{m,L}\left(t\right)=\overset{N^{t}}{\underset{i=1}{\sum }}\overset{M^{t}}{\underset{j=1}{\sum }}W_{ij}^{t,L}\cdot z_{ij}^{t,L}\left(t\right)+\overset{N^{v}}{\underset{i=1}{\sum }}\overset{M^{v}}{\underset{j=1}{\sum }}W_{ij}^{v,L}\cdot z_{ij}^{v,L}\left(t\right).$$

*z*_*ij*_^*s*, *L*^(*t*) (*s* = *t,v*) represents the activity of the unimodal neuron *ij* in tactile or visual area. *W*_*ij*_^*s*, *L*^ denotes the feedforward synapses from the unisensory neuron *ij* to the bimodal neuron; their strength is independent of the position of the unimodal neuron (*W*_*ij*_^*s*, *L*^ = *W*_0_^*s*, *L*^, for all *i*, *j*).

(b) The second contribution is due to the attentional top-down bias input reproduced as a constant input (*I*_bias_^*m*, *L*^). In basal conditions, the attentional bias to the bimodal neuron is set equal to zero in both hemispheres.

The activity of the bimodal neuron (*z* ^*m*,*L*^(*t*)) is obtained from its overall input via a first order dynamics and a static sigmoidal characteristic.

#### 2.2.3. The Inhibitory Interneuron

The inhibitory interneuron in one hemisphere receives synapses from the bimodal neuron in the other hemisphere. Hence, the input to the interneuron in the left hemisphere is

(8) $$u^{g,L}\left(t\right)=X^{R}\cdot z^{m,R}\left(t-D\right),$$

where *z* ^*m*,*R*^(*t*) is the activity of the bimodal neuron in the right hemisphere and *D* is a pure delay, simulating the interhemispheric transit time. *X* ^*R*^ represents the strength of the cross-connection.

Starting from this input, a lowpassdynamics and a sigmoidal static function are used to compute the activity of the interneuron (*z* ^*g*,*L*^(*t*)).

Basal values for all model parameters were assigned on the basis of neurophysiological [21, 25, 26], psychophysical [27, 28], and behavioural literature [29–31] (a detailed parameter assignment can be found in our previous papers [23, 24]). The two hemispheres have the same parameter values. Moreover, unimodal tactile and visual areas in each hemisphere are characterized by the same parameter values (static and dynamic neuron characteristics, lateral synapses).

### 2.3. Simulation Trials Description

To investigate the neural correlates of exogenous/endogenous attention, we simulated the delivery of target stimuli (of both modality and on any side of space) to the hypothetical subject, under four different conditions: the first represents a “neutral”, that is,*unbiased*, condition whereas the other three aim at resembling different conditions of *attentional orienting* towards one side of space. Specifically, we considered the following cases:

- “neutral” condition (*unbiased*): the network works in basal condition, without any unbalance between the two hemispheric subnetworks,

(B) voluntary (*endogenous*) allocation of attention towards one side of space, *irrespective of the modality*: application of a nonzero top-down bias input to the bimodal area in one hemisphere contralateral to the attended side,

(C) voluntary (*endogenous*) allocation of attention towards one side of space, *in a particular modality*, for example, tactile: application of a nonzero top-down bias input to the tactile area and the bimodal area (but not to the visual area) in one hemisphere contralateral to the attended side,

(D) involuntary (*exogenous*) capture of attention towards on side of space: application of a short tactile cue on a specific side (corresponding to the cued side), before any target stimulus; in this condition, the top-down bias input is maintained at zero in both hemispheres.

Both tactile and visual targets are reproduced via a two-dimensional Gaussian function of *x* and *y* coordinates, with small standard deviation to reproduce punctual stimuli as those used in invivo studies. The amplitude of the target stimulus is affected by a Gaussian random noise to create variability in the network response (as it occurs in real conditions). Tactile cue in the exogenous attention condition was simulated as a brief (30 milliseconds) stimulus.

For each condition (A, B, C, D), we performed four blocks of simulations, each block being characterized by the target modality (visual or tactile) and the side of target application (left or right). Ten simulations were performed in each block (40 simulations per condition). Each simulation lasted until the network transient response to the target stimulus was exhausted (see result figures). Network performance in response to each target stimulus was assessed by computing the 98% settling time (*Ts*) of the bimodal neuron on the same side as the target.

## 3. Results

All results describe network response to application of a target stimulus on one side of space (i.e., in each simulation, a single target stimulus on either side is delivered to the network). Each simulation is continued as long as the network reaches a new steady state in response to the target (target application is maintained until the end of the stimulation). Targets are applied while the network operates in one of the four different conditions described above (A, B, C, D).

Table 1 reports the bimodal neuron settling time (mean ± std) as a function of target modality and target side in all examined conditions. Data are analysed via two-tailed paired *t*-tests.

Condition A corresponds to the absence of any specific spatial allocation of attention (“neutral” condition). Simulations in this condition show network basal functioning and provide reference values for network settling time.

**Figure 2:**
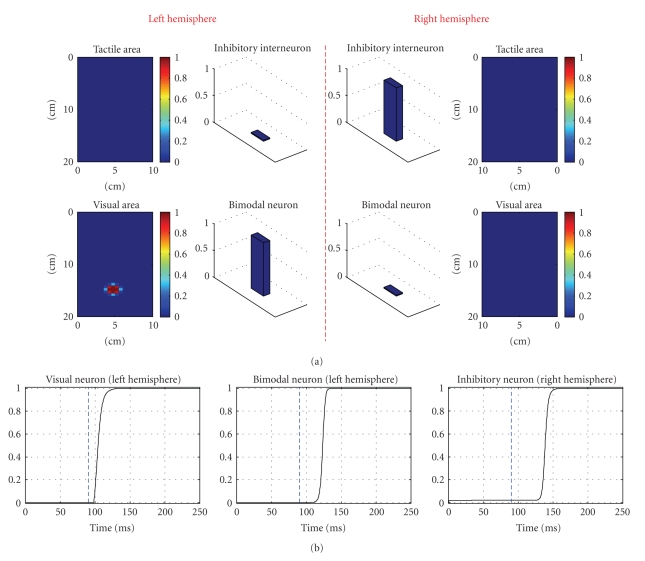
Exemplary network response to a visual target on one hand (the right hand) in basal conditions. Only the response to a visual target on one side is shown, since in condition A the network behaves in a similar way for targets on any side and modality. The target stimulus is maintained, from the onset time of application, throughout the entire simulation. *Panel (a)* shows network activity in steady-state conditions, that is, after the transient response to the stimulus has exhausted. The *x* and *y* axes of the unimodal areas represent the spatial coordinates of neurons RF center; the colour denotes the level of activation of the neurons. *Panel (b)* shows the temporal pattern of the activated neurons; the blue vertical dashed line indicates the onset time of the target stimulus. The left panel displays only the response of the visual neuron on which the stimulus is centred.

Figure 2 displays the neural activity in response to a visual target on one side, in unbiased, neutral condition. Panel (a) shows the steady-state response of the overall network after the transient has exhausted. The visual stimulus induces the activation of a group of neurons in the visual unimodal area; the occurrence of an “activation bubble” is due to the partial superimposition of the RFs of adjacent neurons and to the lateral excitation within the unimodal area which produces reciprocal reinforcement of neighbouring neurons activity. The bimodal neuron in the same hemisphere is activated thanks to the large input from the stimulated visual area. The bimodal neuron, in turn, elicits activation of the inhibitory interneuron in the opposite hemisphere via the interhemispheric synapse. All other neurons in the network are silent. Panel (b) shows the temporal pattern of the activated neurons (precisely, the visual neuron on which the stimulus was centred, the bimodal neuron in the same hemisphere, and the inhibitory interneuron in the opposite hemisphere). The blue dashed vertical line denotes the onset of target application. The visual neuron exhibits a fast response (left plot); whereas the bimodal neuron takes some milliseconds to reach its new steady-state activity following target application (central plot).The inhibitory interneuron follows the same temporal pattern of the contralateral bimodal neuron with few milliseconds delay (right plot), due to the delay in the interhemispheric connection.

Performances of the network in this unbiased condition (see Table 1 Section A) do not differ across sides and modalities (the two hemispheres are perfectly symmetrical, and no difference exists between the tactile and visual areas within each hemisphere).

**Figure 3:**
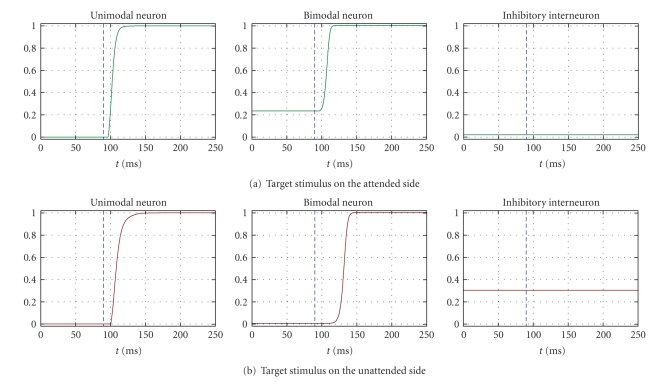
Exemplary temporal patterns of neuron responses to a visual target delivered either on one side or on the other side in condition B (application of the top-down bias input = 10 to the bimodal neuron in one hemisphere contralateral to the attended side). Only the responses to a visual target are shown since in condition B responses on each side do not differ significantly across modalities. *Panel (a)* displays the response of neurons in the attended side to a visual target on that side. *Panel (b) *displays the response of neurons in the unattended side to a visual target on that side. In each panel, the displayed curves are the time response of the unimodal neuron on which the target stimulus is centred, the time response of the bimodal neuron, and the time response of the inhibitory neuron. The blue vertical dashed line denotes the onset time of the target stimulus, maintained throughout the rest of the simulation.

Figure 3 displays the exemplary temporal pattern of neural activity in response to a *visual* target in condition B. In condition B, a top down bias input (*I*_bias_ = 10) is tonically applied to the bimodal neuron in one hemisphere (contralateral to the “attended” side of space). This aims at resembling conditions of voluntary allocation of attention towards one side of space in a supramodal fashion, as it might be necessary when target modality is uncertain. The visual target was applied first on the attended side (panel (a)) and then on the unattended side (panel (b)); that is, the target was present on a single side at a time. In both panels, we displayed the temporal patterns of the unimodal neuron on which the target is centred and of the bimodal neuron and inhibitory interneuron at the same side as the target. The dashed blue vertical line in each plot denotes the time onset of target application. It is worth noticing that the bias input is applied throughout the entire simulation (i.e., even before the target application). The bias input produces a sustained baseline activation of the bimodal neuron in the attended side (up to 20% of its maximum activity) and a consequent tonic activity of the inhibitory interneuron in the opposite hemisphere (corresponding to the unattended side); these effects can be observed, respectively, in Figure 3(a) central panel before target application and in Figure 3(b) right panel. A target applied on the “attended” side produces faster responses of the bimodal neuron thanks to its baseline excitation (compare the response of the bimodal neuron to the target in Figure 3(a) central panel, with respect to unbiased condition in Figure 2(b) central panel). Conversely, a target applied on the “unattended” side is disadvantaged because of the nonnull activity of the inhibitory interneuron (Figure 3(b) right panel) activated by the bias input to the bimodal neuron in the other hemisphere; the inhibitory interneuron slows down the response of the unimodal neurons (Figure 3(b) left panel) and—as a consequence—of the bimodal neuron too (Figure 3(b) central panel). Network performances within each side (attended/unattended) did not differ across modalities (see Table 1); on the contrary, an extremely significant effect of side (attended versus unattended) was found for targets in both modalities (Table 1).

Results of these simulations are consistent with invivo data obtained from subjects who voluntarily allocate their attention towards a specific side of space irrespective of the modality [15]. In that study, participants were informed about the likely side for the upcoming target (towards which they oriented their attention), whereas target modality was uncertain (tactile or visual in every trial): speeded discrimination responses were significantly faster on the expected side than on the other side for targets in both modalities, and the amount of facilitation was similar for visual and tactile targets.

**Figure 4:**
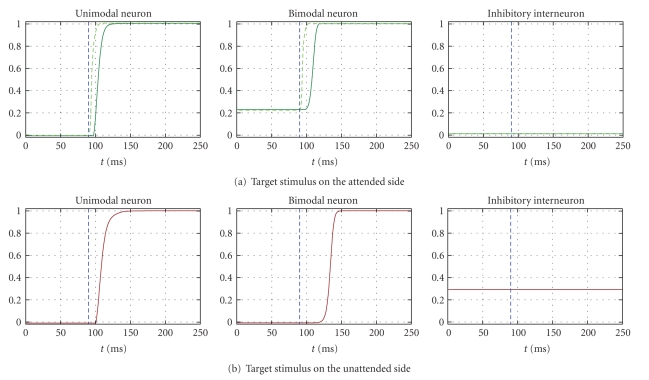
Exemplary temporal patterns of neuron responses to a target delivered either on one side or on the other side in condition C (application of the top-down bias input = 10 to the tactile area and to the bimodal neuron in one hemisphere contralateral to the attended side).* Panel (a)* displays the response of neurons in the attended side to both a tactile target (dashed light green line) and a visual target (continuous dark green line) on that side. On the attended side, network responses to tactile targets are faster than those to visual targets. *Panel (b) *displays the response of neurons in the unattended side to a visual target on that side. In each panel, the displayed curves are the time response of the unimodal neuron on which the target stimulus is centred, the time response of the bimodal neuron, and the time response of the inhibitory neuron. The blue vertical dashed line denotes the onset time of the target stimulus, maintained throughout the rest of the simulation.

In condition C, a top-down bias input (*I*_bias_ = 10) is applied to both tactile and bimodal neurons in one hemisphere (corresponding to the attended side). Figure 4 displays typical network responses to a target applied on the attended side (panel (a)) or on the unattended side (panel (b)) in this condition. In this figure too, each panel shows the temporal response of the unimodal neuron on which the stimulus was centred and of the bimodal neuron and inhibitory interneuron at the same side as the target. In this case, the bias input is applied also to the tactile unimodal area; hence some differences in network response may occur between tactile and visual targets delivered to the attended side. For this reason, in panel (a) we reported neuron responses to both a tactile and a visual target. The dashed blue vertical line in each plot denotes the time onset of target application. The bias input to tactile and bimodal areas in the attended side is applied throughout the entire simulation (even before the target application). As in condition B, the bias input applied to the bimodal neuron produces a tonic activity in the bimodal neuron (see Figure 4(a) central panel, before target application) as well as in the inhibitory interneuron in the opposite hemisphere (see Figure 4(b) right panel). On the contrary, the bias input applied to the tactile neurons is not sufficient to activate them (tactile neurons are silent before target application; see green dashed line in Figure 4(a) left panel); anyway, the bias input leads tactile neurons closer to their excitation threshold. Therefore, when a target is applied on the attended side, the bimodal neuron responds faster because of its baseline excitation due to the bias input. Moreover, since the tactile unimodal neurons are closer to their excitation threshold (thanks to the bias input), their response is more rapidly. Consequently, response performances for tactile targets on the attended side are significantly better than those for visual targets on the same side (see Figure 4(a) left and central panels, and Table 1). Similarly to condition B, responses to targets on unattended side are slowed down because of the tonic activity of the inhibitory interneuron (produced by the bias input to the contralateral bimodal neuron, Figure 4(b)). Significant effects of side application were present for targets in both modalities, although the effect was larger for targets in the tactile modality, because of their higher advantage on the attended side (Table 1).

Network performances in this condition agree with invivo data obtained from subjects who intentionally direct attention in a specific modality towards one side of space [15]. In such experiments, subjects were instructed to direct their attention to one side in just one modality (e.g., tactile, primary modality), without any specific allocation of attention for the secondary modality (e.g., visual). Subjects showed faster discriminations on the side that was attended in the primary modality not only for targets in that modality but also for targets in the secondary modality. Spatial effects were significant for both modalities, although larger for the targets in the primary modality: indeed, on the attended side, targets in the primary modality were more facilitated than targets in the secondary modality [15].

**Figure 5:**
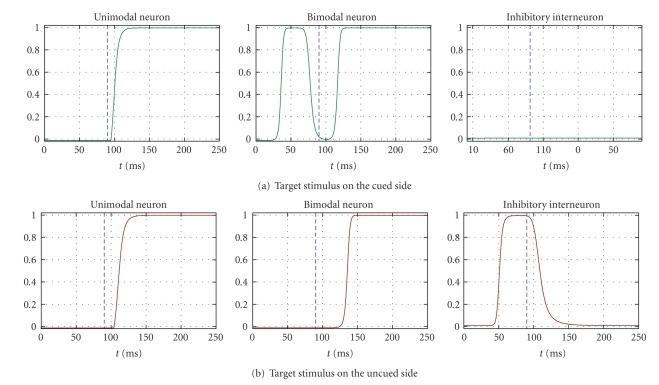
Exemplary temporal patterns of neuron responses to a visual target delivered either on one side or on the other side in condition D (a tactile cue of 30 milliseconds preceded any target stimulus; stimulus onset asynchrony = 90 milliseconds). Only the responses to a visual target are shown since, in condition D, responses on each side do not differ significantly across modalities. *Panel (a)* displays the response of neurons in the cued side to a visual target on that side. *Panel (b)* displays the response of neurons in the uncued side to a visual target on that side. In each panel, the displayed curves are the time response of the unimodal neuron on which the target stimulus is centred, the time response of the bimodal neuron, and the time response of the inhibitory neuron. The blue vertical dashed line denotes the onset time of the target stimulus, maintained throughout the rest of the simulation.

In condition D, a 30-millisecond tactile cue is delivered to one side (“cued” side), before the application of any target stimulus (stimulus onset asynchrony = 90 milliseconds). The top-down input is set to zero in both hemispheres. Exemplary responses to a visual target on the cued (panel (a)) or uncued (panel (b)) side are shown in Figure 5. Each panel shows the temporal response of the unimodal neuron on which the stimulus was centred and of the bimodal neuron and inhibitory interneuron at the same side as the target. The blue dashed vertical line in each plot denotes the onset of target application. The transient response of the bimodal neuron in the cued side before target onset (see Figure 5(a) central plot) is elicited by the tactile cue. Analogously, the transient response of the inhibitory interneuron in the uncued side (Figure 5(b) right plot) is due to the application of the tactile cue on the cued side, via the interhemispheric synapse. Responses were faster for targets (both modalities) on the same side as the cue (see Table 1): indeed, at the time of target application, the bimodal neuron on the cued side has not completely recovered its baseline condition (Figure 5(a) central panel) and it is closer to the threshold level. Conversely, the response to a target on the uncued side is hindered by the inhibitory action of the interneuron, transiently activated by the cue on the opposite side. In particular, because of the interhemisheric delay, the inhibitory interneuron on the uncued side is still active at the target time presentation (Figure 5(b), right panel), slowing down the response of the unimodal neurons and, consequently, of the bimodal neuron (Figure 5(b), left and central panels). On the overall, network responds significantly more rapidly on the cued side than on the uncued side, for targets in both modalities, not only in the cue modality.

Network performances are in agreement with results of exogenous attention studies [13]. In the study by Kennett et al. [13], single visual stimuli presented on the left or right hand were preceded by a spatially nonpredictive tactile cue; responses to visual targets were faster and more accurate when these were presented on the cued versus the uncued side.

## 4. Discussion

Many behavioral and neuroimaging studies have revealed that attention operates across different sensory modalities [12, 13, 15, 16, 19]. Despite considerable experimental research, understanding the neural basis of cross-modal links in spatial attention still remains a significant challenge to cognitive neuroscience. In this work, we used a neural network model to elucidate the mechanisms and possible neural correlates of multisensory integration in spatial attention.

Neurocomputational models have been proposed in literature to explain mechanisms of unimodal attention. Most of these models aimed at investigating different aspects of visual attention [6, 9, 32, 33]; recently, artificial neural networks have been applied also to study attentional effects in other modalities too (e.g., somatosensory) [34]. At present, we are not aware of any work that exploits a computational modelling approach in order to shed light on the neural mechanisms underlying multimodal spatial attention.

We implemented a simple neural network with limited complexity, which includes two unimodal areas and a bimodal area connected via excitatory feedforward and feedback synapses within each hemisphere and a competitive interaction via inhibitory interneurons between the two hemispheres.

Such model architecture has several physiological counterparts. The bimodal neurons in the model may represent cells in the parietal and frontal cortex that have been found to respond to tactile stimuli on a specific body part (e.g., the hand) and to visual stimuli near the same body part [20–22]. The visual and tactile receptive fields of such neurons are in close spatial register and can be very large, even encompassing the entire hand [21, 22]. Some studies [13, 35] suggested that such bimodal neurons may be involved in generating the visual-tactile links in spatial attention. The two upstream unimodal layers in the model account for primary and secondary somatosensory and visual areas, which project into the multisensory areas through different pathways [20–22]. The presence of back-projections from the bimodal neuron into the upstream unimodal areas is supported by neuroimaging data according to which activity in the tactile area due to a tactile stimulus is amplified by a concurrent visual stimulus on the same hand, and vice versa [36, 37]. However, in the simulations performed in the present study, feedback connections do not play any role, since we applied only one unimodal stimulus at a time on each hand.

Several researches (see [38] for a review) suggest that the functional interaction between the two hemispheres may be inhibitory at times and excitatory at other times, depending on the task. In some instances, it may be more efficient for the hemispheres to compete, so that the dominant hemisphere takes the control of the processing; in other instances, interhemispheric cooperation might be necessary to complete the task. In particular, the existence of an interhemispheric competition for accessing limited attentional resources has received several neurophysiological and behavioural evidence [1, 31]. The most striking evidence of attentional competition between the hemispheres is provided by right brain damaged patients, suffering from sensory extinction. They are able to detect stimuli presented to either side of space but fail to detect the stimulus on the contralesional side when both sides are stimulated simultaneously, even under two different sensory modalities (e.g., one stimulus is visual and the other tactile [39]). An attention-capturing event on the ipsilesional intact side competes with—and may completely extinguishe—the stimulus on the contralesional affected side.

Thanks to this network structure, we are able to reproduce several phenomena of cross-modal interactions in both endogenous (voluntary or top-down) and exogenous (reflexive or bottom-up) attention. It is worth noticing that to relate model results with behavioural responses (i.e., reaction times), network performances have been evaluated in terms of bimodal neuron settling time, rather than unimodal neurons response. Indeed, recent studies [40, 41] suggest that activation of early sensory cortices is not sufficient to produce perceptual awareness; rather, the conscious perception of sensory stimuli depends on the activation of higher level multimodal areas (in a parietal-frontal network), which make conscious information available for further processes such as memorization, action, and verbal report [42].

In order to simulate endogenous allocation of attention, we introduced an external top-down bias, that is, an excitatory input to pools of neurons in the hemisphere contralateral to the attended side of space. Indeed, it has been argued [43, 44] that the direction of covert spatial attention depends on the relative level of activation between the two cerebral hemispheres, so that higher left hemisphere activation tends to direct attention rightwards and viceversa. Moreover, experimental and theoretical studies on visual system indicate that attention may act as an excitatory input boosting the activity of neurons encoding the attended stimuli [6, 32, 45]. Crucially, our model analysis shows that boosting activity in appropriate brain areas via top-down bias input may explain various—and for some aspects controversial—results of endogenous attention.

By applying the top-down bias input to just the bimodal neuron in one hemisphere, the model predicts an improvement in the perception of contralateral stimuli (in the attended side) and shows that such improvement generalizes with the same extent for stimuli in both modalities. Such results are in agreement with invivo data obtained when only target location is known in advance and target modality is unpredictable [15]. In this case, since the target modality is uncertain, attentional boosting of only a supramodal system would have both functional and parsimonious significance.

By applying the top-down bias input to the bimodal neuron and to a pool of unimodal neurons (e.g., tactile) in the same hemisphere, the network predicts that performance improvement on the attended side for targets in the biased modality (tactile) is larger than for targets in the unbiased modality (visual), as observed experimentally when attending to a spatial location in a specific modality [15]. Hence, attentional boosting of both a supramodal system and a modality-specific system can explain how endogenous attention in a primary modality can spread into other modalities, but only in an attenuated fashion. Conversely, if the biased input was applied only to one area of unimodal (e.g., tactile) neurons, the model would not be able to reproduce the spreading of attention into the other modality (unshown simulations). Model postulations are supported by recent neuroimaging studies showing that sustained spatial attention within one modality modulates the activity of both modality-specific and multimodal areas (e.g., in the intraparietal sulcus) [18].

Hence, model analysis suggests that endogenous attention may operate entirely at a supramodal level or at both modality-specific and supramodal levels, depending on whether subject's expectancy concerns only stimuli location or also stimuli modality.

By setting the top-down bias input at zero and presenting a cue before any target, the model is able to reproduce results of exogenous attention [13]. In the model, capture of attention both in the cue modality and in the other modality is due to the involvement of the bimodal neuron, which receives advantage activation thanks to the cue stimulus.

An important aspect of simulation results is that both in endogenous and in exogenous attention, the network reproduces not only response benefits at the attended/cued side but also response detriments at the unattended/uncued location [46] (see also [47], for a review). Detrimental responses at the unattended/uncued side arise from the competitive interaction between the two hemispheres, so that the increased activity in the attended side hemisphere (due to the bias input or to the cue) inhibits activity in the opposite hemisphere.

To sum up, differences in network performances between the two sides (attended versus unattended or cued versus uncued) are mediated by the same mechanisms in both endogenous and exogenous attention. (1) The shift of the working point of the bimodal neuron above or near the threshold of its activation function, induced by the top-down bias or by the cue application, speeds up the responses to targets on the attended/cued side. This facilitation may be further improved in one modality, by adding a further bias to a unimodal area. (2) The tonic/phasic activation of the inhibitory interneuron on the unattended/uncued side (due, resp., to the contralateral top-down bias or cue application), slows down the responses to targets on that side.

Hence, our study shows that the unimodal and cross-modal effects of orienting attention to one hemispace both exogenously and endogenously can be explained via a model of cooperative and competitive interactions among unimodal and bimodal areas in the two hemispheres: interhemispheric interactions can be biased both by a stimulus-driven or by a top-down mechanism. According to our results, the Biased Competition Hypothesis, proposed in the context of the visual system and successfully explaining several aspects of visual attention, applies also to crossmodal attention, suggesting that a basic mechanism of attention may be replicated at different levels and across multiple areas and multiple sensory modalities in the brain. The novel insight coming from the present results is that spatial attentional orienting acting both at a unimodal level and at a multimodal level seems to necessarily tap onto associative, multisensory brain areas, probably located in the parietal cortex.

The proposed model may be of value to help interpretation of behavioural results on spatial attention and to drive experiments on attention and cross-modal construction of space. Future studies may be devoted to investigate further aspects not considered in the present work, such as the “inhibition of return” effect [48].

## Figures and Tables

**Table 1:** Settling time (*Ts*) of the bimodal neuron (mean ± std) in response to tactile and visual targets on both sides, in the four examined conditions, and results of two-tailed paired *t*-tests on tactile-minus-visual differences and unattended/uncued side-minus-attended/cued side differences (ns: nonsignificant: *P < .05; **P < .01; ***P < .01).

Condition A			
	One side	Other side	Difference
Tactile	*Ts* = 41.9 ± 1.7 ms	*Ts* = 41.5 ± 3.9 ms	Δ = −0.44 ± 3.7 ms(ns)
Visual	*Ts* = 39.8 ± 3.3 ms	*Ts* = 40.3 ± 4.0 ms	Δ = 0.48 ± 4.7 ms (ns)
Difference	Δ = 2.12 ± 3.4 ms (ns)	Δ = 1.2 ± 4.86 ms (ns)	

Condition B			

	Attended side	Unattended side	Difference
Tactile	*Ts* = 24.1 ± 1.1 ms	*Ts* = 52.5 ± 11 ms	Δ = 28.36 ± 11 ms***
Visual	*Ts* = 25.4 ± 3.8 ms	*Ts* = 54.8 ± 13 ms	Δ = 29.44 ± 12.5 ms***
Difference	Δ = −1.32 ± 3.7 ms(ns)	Δ = −2.4 ± 18.5 ms(ns)	

Condition C			

	Attended side	Unattended side	Difference
Tactile	*Ts* = 10.6 ± 0.7 ms	*Ts* = 56.4 ± 8 ms	Δ = 45.8 ± 8.6 ms***
Visual	*Ts* = 26.7 ± 1.6 ms	*Ts* = 53 ± 11 ms	Δ = 26.3 ± 11.6 ms***
Difference	Δ = −16.2 ± 2.11 ms***	Δ = 3.4 ± 13.4 ms (ns)	

Condition D			

	Cued side	Uncued side	Difference
Tactile	*Ts* = 32.9 ± 1.7 ms	*Ts* = 58.5 ± 6.9 ms	Δ = 25.6 ± 8.1 ms***
Visual	*Ts* = 34.1 ± 1.2 ms	*Ts* = 56.1 ± 9.3 ms	Δ = 22 ± 10.3 ms***
Difference	Δ = −1.2 ± 2.16 ms(ns)	Δ = 2.4 ± 12.5 ms(ns)	
